# Adversarial Multiscale Feature Learning Framework for Overlapping Chromosome Segmentation

**DOI:** 10.3390/e24040522

**Published:** 2022-04-07

**Authors:** Liye Mei, Yalan Yu, Hui Shen, Yueyun Weng, Yan Liu, Du Wang, Sheng Liu, Fuling Zhou, Cheng Lei

**Affiliations:** 1 The Institute of Technological Sciences, Wuhan University, Wuhan 430072, China; liyemei@whu.edu.cn (L.M.); wengyueyun@whu.edu.cn (Y.W.); wangdu@whu.edu.cn (D.W.); victor_liu63@vip.126.com (S.L.); 2 The Department of Hematology, Zhongnan Hospital of Wuhan University, Wuhan 430071, China; yuyalan@znhospital.cn (Y.Y.); shenhui@znhospital.cn (H.S.); zhoufuling@whu.edu.cn (F.Z.); 3 The Key Laboratory of Transients in Hydrolic Machinery of Ministry of Education, School of Power and Mechanical Engineering, Wuhan University, Wuhan 430072, China; 4 The Alipay Tian Qian Security Lab., Beijing 100020, China; goodmandou@gmail.com

**Keywords:** overlapping chromosome segmentation, conditional generative adversarial network, nested U-shaped network, multiscale feature learning, Lovász-Softmax

## Abstract

Chromosome karyotype analysis is of great clinical importance in the diagnosis and treatment of diseases. Since manual analysis is highly time and effort consuming, computer-assisted automatic chromosome karyotype analysis based on images is routinely used to improve the efficiency and accuracy of the analysis. However, the strip-shaped chromosomes easily overlap each other when imaged, significantly affecting the accuracy of the subsequent analysis and hindering the development of chromosome analysis instruments. In this paper, we present an adversarial, multiscale feature learning framework to improve the accuracy and adaptability of overlapping chromosome segmentation. We first adopt the nested U-shaped network with dense skip connections as the generator to explore the optimal representation of the chromosome images by exploiting multiscale features. Then we use the conditional generative adversarial network (cGAN) to generate images similar to the original ones; the training stability of the network is enhanced by applying the least-square GAN objective. Finally, we replace the common cross-entropy loss with the advanced Lovász-Softmax loss to improve the model’s optimization and accelerate the model’s convergence. Comparing with the established algorithms, the performance of our framework is proven superior by using public datasets in eight evaluation criteria, showing its great potential in overlapping chromosome segmentation.

## 1. Introduction

**Figure 1:**
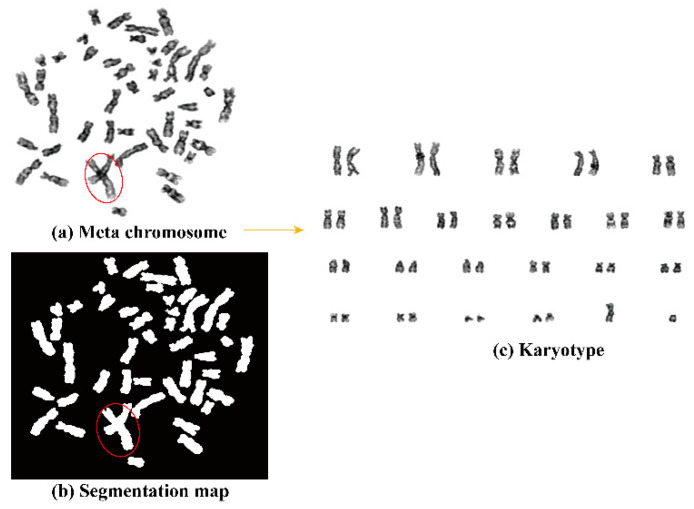
Chromosome karyotype analysis process. (**a**) The image of meta chromosome. (**b**) Segmentation map of the chromosome image. (**c**) The sorted karyotype. The red circles mark the overlapping regions.

Human chromosome karyotype analysis is of great diagnostic and prognostic value in diseases. It is usually performed in clinical diagnosis, cancer cytogenetics, and the detection of genetic abnormalities such as Edwards syndrome and Down syndrome [1,2]. The morphology of chromosomes, such as extra or missing chromosomes, or the structural defects of specific chromosomes can be directly linked to corresponding diseases; hence, chromosome karyotype analysis based on images plays a critical role in routine disease diagnosis and treatment [3]. Figure 1 illustrates the process of chromosome karyotype analysis, which consists of two main steps: segmentation and classification. The performance of the segmentation can directly influence the accuracy of the classification afterward. Although single chromosome classification could achieve high accuracy [4]. However, as shown in the red circles in Figure 1a,b, for the overlapping chromosome segmentation is even more challenging due to the ambiguity in the overlapping regions, which can greatly influence the accuracy of the chromosome karyotype analysis. Hence, in this study we mainly focus on pushing the limit removing the restrictions of overlapping chromosome segmentation and designing a highly efficient and accurate overlapping-chromosome segmentation method to enhance the overall performance of the chromosome karyotype analysis.

Since manual segmentation is both time and effort consuming and the accuracy highly depends on the experience level of the analyst, over the past few decades, many algorithms have been proposed to automatically segment the chromosome from the images on computers [5]. Based on their principles, these segmentation methods can be roughly classified into two categories: heuristic methods and learning-based methods. Heuristic methods utilize manually tagged features, such as contour, pixels, and geometric features, to perform segmentation [6,7,8]. Specifically, Ritter et al. utilized shape analysis and classification for chromosome segmentation and adopted global context and variant analysis methods to solve complex and ambiguous cases [9]. However, it consists of two phases and is somewhat cumbersome. Madian et al. used the contour analysis method and constructed reasonable hypotheses for segmentation and separation [10]. Saiyod et al. proposed an edge detection method that consisted of the flood fill, erosion, and canny methods [11]; however, it only solved the touching chromosomes and not the overlapping chromosomes. Some researchers usually use thresholding strategies for chromosome segmentation [12,13,14], adopting a local adaptive histogram equalization technique to obtain the appropriate threshold, to further enhance chromosome segments by reducing the chances of pixel misclassification. However, these methods are susceptible to noise, contrast, and poor resolution of the image. Gawande et al. applied a fuzzy C-means clustering algorithm and watershed algorithm for chromosome segmentation, but it also did not effectively separate overlapping chromosomes [15]. Sharma et al. adopted a combination of crowdsourcing for segmentation, but it required considerable effort and time to separate chromosomes manually [16]. Lin et al. proposed a geometric feature to separate chromosomes [17]; however, it could not automate chromosome segmentation well due to the irregular shape of chromosomes. These methods can achieve impressive segmentation results when manual features are properly tagged; however, they are very sensitive to the shape and overlapping regions of the chromosomes. Moreover, since they do not consider the untagged features, the performance and applicability of these methods are limited, and it is difficult to implement them on large datasets. Meanwhile, Learning-based methods usually applied machine learning techniques to mine potential information from the images to perform chromosome segmentation and medical image analysis [18,19]. Some representative examples include Pardo et al., who applied the fully convolutional network (FCN) method for karyotype analysis [20,21]. However, it does not contain overlapping chromosomes, therefore it was difficult to meet the clinical practice. Other researchers use a U-shaped Network (UNet) for overlapping chromosome segmentation [22,23]. Chen et al. proposed the shape learning method to segment both non-overlapped and overlapped regions [24]. Altinsoy et al. proposed a raw G-band chromosome image segmentation method using convolution network [25], but it did not work for overlapping chromosomes. These methods can independently conduct chromosome segmentation when being trained. However, limited by the architecture of the network, current learning-based methods only utilize several layers’ features, and they do not take advantage of multiscale features to adapt different chromosome scales. Hence, they do not perform well in overlapping chromosome segmentation. Recently, Chen et al. proposed a multiscale adversarial network [26] for fine-grained image categorization and achieved good classification performance. This provides a new way of thinking about our work.

In this paper, considering the various scales and overlapping regions of chromosome images, we demonstrate an adversarial multiscale feature learning (AMFL) framework that employs a nested U-shaped convolutional neural network (NestedUNet) [27], conditional generative adversarial network (cGAN) [28], and Lovász-Softmax [29] for overlapping chromosome segmentation. Specifically, NestedUNet consists of UNets [30] of varying depths and owns dense skip connectivity, making it capable of synthesizing multiscale feature maps for segmentation. Hence, our AMFL framework utilizes NestedUNet to explore the optimal representation of chromosome images by exploiting multiscale features and fused features. Moreover, we consider the overlapping chromosome image segmentation as an images-to-image task, in which the source overlapping chromosome images are translated to a confidence map to indicate the category information in the source images; we, therefore, use cGAN to push the output distributions close to the ground truth for its success in computer vision tasks, such as image deblurring [31] and image fusion [32]. Finally, to optimize the performance of the discriminatively trained overlapping chromosome segmentation, we apply Lovász-Softmax, which is based on the convex Lovász extension of the submodular loss, as the segmentation loss to achieve superior chromosome segmentation performance and higher index scores compared to the traditional cross-entropy (CE) loss. Additionally, we utilize the least-square GAN objective [33] to replace the original GAN loss in the overlapping chromosome segmentation task to stabilize the training and avoid model collapse. To verify the feasibility of our method, we carry out extensive experiments to compare the performance of our AMFL framework with others. Results show the superiority of our AMFL framework in this work in terms of visual perception analysis and quantitative score comparison. The major contributions of this paper are summarized as follows:

1. We present an adversarial multiscale feature learning framework to improve the accuracy and adaptability of overlapping chromosome segmentation.
2. We use the conditional generative adversarial network to penalize the difference between the generated decision map and the source image, pushing the generator to produce a higher-confidence decision map for the segmentation task.
3. Instead of using the single-scale features to represent the chromosome images, we carefully design a nested U-shaped network with dense skip connections as the generator to capture multiscale features to explore the better representation of the chromosome images.
4. We replace the common cross-entropy loss with the advanced Lovász-Softmax loss to improve the model’s optimization and accelerate the model’s convergence.
5. We carry out extensive experiments and analyze different objective functions that provided baselines for chromosome segmentation.

Results show the superiority of the present AMFL method and the loss function adopted in this work in terms of visual perception analysis and quantitative score comparison.

## 2. Materials and Methods

### 2.1. Network Architecture

**Figure 2:**
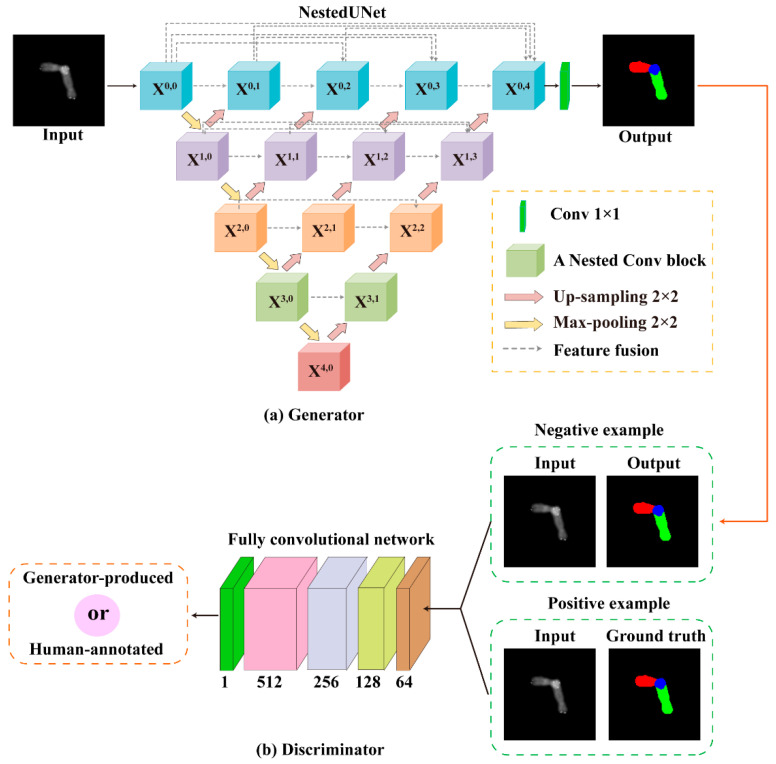
The pipeline of the proposed framework, which consists of two components: (**a**) a generator and (**b**) a discriminator. The generator receives a chromosome image as input and outputs a fake segmentation map, and the discriminator attempts to distinguish it from the real ground truth.

In this paper, we consider the overlapping chromosome image segmentation as an image-to-image task, in which the source overlapping chromosome images are translated to a confidence map to indicate the category information in the source images. Compared with the existing methods, we bring the adversarial learning to penalize difference between the generated decision map and source image, pushing the generator to produce a higher-confidence decision map for segmentation task. Moreover, the generator in our framework is deeper than that of CNN-based methods, possessing higher representational capacity. Specifically, as depicted in Figure 2, similar to the original GAN [28], our AMFL framework consists of two modules: a generator and a discriminator. The generator is responsible for exploiting multiscale features for segmentation by producing “fake” chromosome images. While the discriminator serves to distinguish the “fake” images from the “real” ones by adversarial learning. Once the discriminator is “fooled” by the generator, the network is ready to segment chromosomes with high accuracy.

(1) Generator: As shown in Figure 2a, we adopted the advanced NestedUNet as the generator G, which consists of an encoder and a decoder. It takes a source chromosome image as the input and outputs a multiclass one-hot map. Specifically, each node in the graph represents a nested convolution block; the downward arrows, upward arrows, and dotted arrows indicate 2 × 2 max-pooling, 2 × 2 up-sampling, and skip connections, respectively. The skip connections merge the encoding and decoding features in the channel dimension by tensor concatenation, enabling dense feature propagation. To better comprehend the network connectivity, we denoted as $x^{i,j}$ the output of the node $X^{i,j}$. It can be formulated in Equation (1):

(1) $$x^{i,j}=\{\begin{array}{l} N(D\left(x^{i-1,j}\right),\;\;\;\;\;\;\;\;\;\;\;\;\;\;\;\;\;\;\;\;\;\;\;\;\;\;\;\;\;\;j=0\\ N\left(\left[{\left[x^{i,k}\right]}_{k=0}^{j-1},U\left(x^{i+1,j}-1\right)\right]\right),j>0 \end{array}$$

where function N(⋅) denotes a nested convolution block operation; D(⋅) and U(⋅) denote a down-sampling layer and an up-sampling layer, respectively; and [⋅] denotes the concatenation layer. Intuitionally, we can see that the nodes at the level of *j* = 0 receive only one input from the previous layer of the encoder, whereas the nodes at the level of *j* > 1 receive the up-sampled output of *j* + 1 nodes from the lower skip connection and all the outputs of the previous *j* nodes in the same skip connection. Therefore, a dense skip connection was constructed and multiscale features are integrated to provide better feature representation for the segmentation of overlapping chromosome regions with different scales. Meanwhile, in order to better describe network parameters, the number of filters was defined as: f={64, 128, 256, 512, 1024}, and the number of input channels, middle channels, and output channels of a nested convolution block were defined as follows:

(2) $$\begin{array}{l} I_{ij}=\left\{ \begin{array}{l} f(i),\;\;\;\;\;\;\;\;\;\;\;\;\;\;\;\;\;\;j=0\\ f(i)\cdot j+f(i),\;\;j>0 \end{array}\right.\\ M_{ij}=f(i),\;\;\;\;\;\;\;\;\;\;\;\;\;\;\;\;\;O_{ij}=f(i) \end{array}$$

In Equation (2), $I_{ij}$, $M_{ij}$, $O_{ij}$ are the input channels, middle channels, and output channels of the *ij*th node, respectively. Note that the middle channels are the output channel of the first convolution layer and also the input channel of the second convolution layer in the nested convolution block. To describe the network structure in detail, we denote the convolution layer, batch norm layer, and rectified linear unit [34] as Conv, BN, and ReLu, respectively. The nested Conv block is two Conv-BN-ReLu layers with a filter size of 3 × 3, padding of 1, and stride of 1, which aims to keep the size of the feature map consistent after each convolution operation. The last layer of convolution kernel size with 1 × 1, and the feature map of the last nodeis mapped into a confidence map using the Softmax operation for producing a one-hot map.

(2) Discriminator: As shown in Figure 2b, inspired by the PatchGAN in [28], we used a simplified fully convolutional neural network [21,32] as the discriminator D, which is able to push the output’s distribution closer to the ground truth, making the generator produce high-confidence segmentation maps. This discriminator tries to distinguish whether each K × K patch in an image is real or fake, and then averages all responses convolutionally across the image to provide the ultimate output of D. Specifically, it consists of five convolution layers with a filter size of 4 × 4 kernel and {64, 128, 256, 512, 1024} channels. The first four convolution layers with padding of 2 and stride of 2, and the last two layers with padding of 2 and stride of 1. Each convolution layer is followed by a Leaky-ReLu parameterized by 0.2 except the last layer. Then a sigmoid function follow with the last layer and can produce a binary output for discriminating “real” or “fake” images. Finally, it is worth noting that the input of the discriminator is multichannel images created by concatenating the source images and the segmented images (generator produced) in the channel dimension, aiming to provide prior information for better-discriminating features. The generator and discriminator were alternately trained using the objective function represented as follows.

### 2.2. Objective Function

(1) Lovász-Softmax: It can optimize the Jaccard index in the continuous optimization framework [29]. Specifically, this method can substantially improve the accuracies of semantic segmentation by optimizing the correct loss during training. Therefore, we chose Lovász-Softmax as the loss of the generator, which can be simplistically defined in Equation (3):

(3) $$L_{Lovász-Softmax}=\frac{1}{C}\sum_{c\in C}{\bar{\Delta J}}_{c}(m(c))\bar{\Delta }$$

where m(c) is a vector of pixel errors for class c∈C aiming to construct the loss surrogate to ${\bar{\Delta J}}_{c}$, it is defined by:

(4) $$m(c)=\left\{ \begin{array}{l} 1-f_{i}(c)\;\;\;\;\;\;\;\;\;\;\;\;\;\mathrm{if}\;\;c=y_{i}^{}\\ f_{i}(c)\;\;\;\;\;\;\;\;\;\;\;\;\;\;\;\;\;\;\;\;\;\;\;\;\mathrm{otherwise} \end{array}\right.$$

In Equation (4), *y* is ground truth, and $f_{i}(c)$ is the predicted scores of the model that is mapped to probabilities through a Softmax unit. $\Delta J_{c}$ is the set function encoding a submodular Jaccard loss for class *c*, indicating a set of mispredictions. Specially, $\bar{\Delta }$ is the surrogate for the minimization of Δ with first-order continuous optimization, and the elementary operations involved in the calculation of Δ *(sort)* are differentiable.

(2) GAN loss: First and foremost, we needed to choose an appropriate loss function for training our AMFL framework. It is well known that the regular GAN [35] loss is always difficult to converge and can suffer from model collapse. We, therefore, adopted the least-squares generative adversarial network (LSGAN) [33] as the loss function in our work, which is more stable and can achieve better segmentation results by previous experimental experience [29]. It is defined by Equation (5):

(5) $$L_{LSGAN}(D)=E_{i,y∼}P_{\mathrm{data}(i,y)}\left[{\left(D(i,y)-1\right)}^{2}\right]+E_{i∼}P_{\mathrm{data}(i)}\left[{\left(D(i,G(i))\right)}^{2}\right]$$

The adversarial learning process is also optimized through the LSGAN, which is formulated in Equation (6):

(6) $$L_{LSGAN}(G)=E_{i∼}P_{\mathrm{data}(i)}\left[{\left(D(i,G(i))-1\right)}^{2}\right]$$

Furthermore, in order to make the segmentation map as close as possible to the ground truth, we adopted Lovász-Softmax loss for supervised segmentation. The objective function for AMFL, therefore, can be defined by Equation (7):

(7) $$\begin{array}{l} \underset{D}{\min}L(D)=L_{LSGAN}(D)\\ \underset{G}{\min}L(G)=L_{LSGAN}(G)+\lambda L_{Lovász-Softmax} \end{array}$$

where λ controls the relative importance of the two objective functions. Empirically, we set λ to 10 in our work.

### 2.3. Evaluation Metrics

To quantitatively evaluate the performance of our method, we selected eight evaluation metrics including the pixels accuracy (Acc) [36], dice coefficient (Dice) [37], intersection over union (IoU) [36], precision, recall, false-negative rate (FNR), false-positive rate (FPR) [36], and Hausdorff distance (Hausdorff) [36], which are briefly introduced below. For convenience, we used ***O*** to denote the output segmentation image and ***G*** to indicate the ground truth. Moreover, the index ranges over the interval [0, 1] except Hausdorff.

(1) *Acc*: It indicates the pixel accuracy of the predicted results in the segmentation. In other words, it represents the proportion of pixels in an image that is correctly predicted. The Acc is calculated using Equation (8):

(8) $$Acc=\frac{\sum_{i=0}^{c}P_{ii}}{\sum_{i=0}^{c}\sum_{j=0}^{c}P_{ij}}$$

Here, $P_{ij}$ means the numbers of pixels that are classified as pixel *j* but actually belongs to pixel *i*, and *c* is the categories.

(2) *Dice*: This metric represents the similarity of the predicted image ***O*** to the ground truth ***G***. The Dice is calculated using Equation (9):

(9) $$Dice_{c}=2\frac{\left|G_{c}\cap O_{c}\right|}{\left|G_{c}|+|O_{c}\right|}$$

where |***G***| and |***O***| represent the numbers of elements in the arrays.

(3) *IoU*: This metric represents the intersection area between the predicted image ***O*** and the ground truth ***G***, the *IoU* can be calculated using Equation (10):

(10) $$IoU_{c}=\frac{\left|G_{c}\cap O_{c}\right|}{\left|G_{c}\cup O_{c}\right|}$$

(4) *Precision*: It indicates how reliable the prediction is. This metric can be calculated using Equation (11):

(11) $$Precsion_{c}=\frac{TP_{c}}{TP_{c}+FP_{c}}$$

where $TP_{c}$ represents the true positives, which means the pixels correctly predicted to belong to class *c*, while $FP_{c}$ represents the false positives, indicating the pixels that are predicted as class *c* but do not actually belong to class *c*.

(5) *Recall*: It indicates how sensitive the prediction is. Therefore, it is also called sensitivity, which can be calculated using Equation (12):

(12) $$Recall_{c}=\frac{TP_{c}}{TP_{c}+FN_{c}}$$

where the $FN_{c}$ represents the false negatives, meaning the pixels that are predicted as not class *c* but actually belong to class *c*.

(6) *FNR*: It is also called the under-segmentation rate, which measures the proportion of the positive classes that are predicted to be negative. It is defined as in Equation (13):

(13) $$FPR_{c}=\frac{FP_{c}}{FP_{c}+TN_{c}}$$

(7) *FPR:* It is also called the over-segmentation rate, measuring the proportion of the negative classes that are predicted to be positive. This metric is calculated using Equation (14):

(14) $$FPR_{c}=\frac{FP_{c}}{FP_{c}+TN_{c}}$$

where $TN_{c}$ represent the true negatives, which mean the pixels that are correctly predicted not to belong to class *c*.

(8) *Hausdorff:* It represents the shape similarity between the predicted images ***O*** and the ground truths ***G***. It is calculated using Equation (15):

(15) $$Hausdorff_{c}=\max\left\{\underset{x\in G_{c}}{\sup}\underset{y\in O_{c}}{\inf}d\left(x,y\right),\underset{y\in S_{c}}{\sup}\underset{y\in G_{c}}{\inf}d\left(x,y\right)\right\}$$

where d(⋅) represents the Euclidean distance between the pixel points *x* and *y*. The smaller the Hausdorff distance is, the greater the similarity between the predicted segmentation maps and the ground truth is.

Note that, for each metric, a higher value indicates better performance, except for *FNR*, *Hausdorff*, and *FPR*, where a lower score gives a better segmentation result

### 2.4. Baselines and Implementation

We validate the effectiveness of our method by comparing it with 10 recent state-of-the-art algorithms, including efficient neural network (ENet) [38], bilateral segmentation network version 1 (BiSeNetV1) [39], BiSeNetV2 [40], DeepLabV3+ [41], faster fully convolutional network (FastFCN) [42], U-shaped network [30] (UNet), recurrent residual UNet (R2UNet) [43], attention UNet (AttUNet) [44], recurrent residual attention UNet (R2AttUNet) [45], and nested UNet (NestedUNet) [27]. Among them, ENet, BiSeNetV1, and BiSeNetV2 are small-scale models that usually have smaller network scales and higher inference speed. While the others are large-scale models that usually have more complex network structures and can learn more potential semantic features. The above methods were used as baselines to evaluate the performance of our method comprehensively. Furthermore, in order to verify our selection of loss function, four commonly used loss functions were tested. Since all the above methods have not been used for the chromosome segmentation task, we instead implemented all the methods ourselves with the same hyperparameters to have a fair comparison.

### 2.5. Selection of the Objective Function and Generator

#### 2.5.1. Selection of the Objective Function

**Figure 3:**
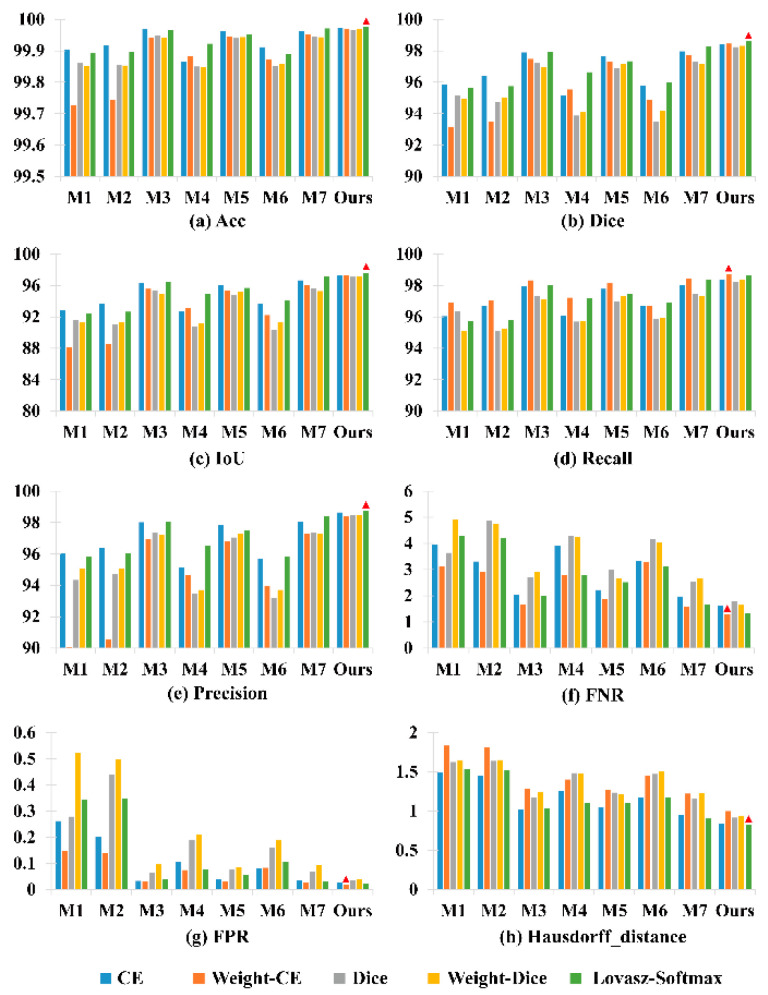
The average quantitative metrics score of different objective loss functions of various methods. The coordinate scale M1–M7 represents DeepLabV3+, FastFCN, UNet, R2UNet, AttUNet, R2AttUNet, and NestedUNet, respectively. The best results are highlighted by the red triangle.

In order to show the superiority of using Lovász-Softmax as the loss for overlapping chromosome segmentation, as shown in Figure 3, we drew average metric graphs for prevalent losses on all testing sets with all the methods. Intuitively, it can be seen that our method with Lovász-Softmax outperformed all baseline methods in all the metrics. Moreover, it is clear that all baseline methods with Lovász-Softmax also show a leading scoring trend against other losses, indicating that Lovász-Softmax is effective and optimal for overlapping chromosome segmentation feature extraction.

#### 2.5.2. Selection of the Generator

In Table 1, we evaluate the performance of our method with different generator networks in terms of all the quantitative indicators. Obviously, we can clearly see that the framework with NestedUNet as the generator network is better than other configurations, indicating that the modeling combined with multiscale features is effective.

### 2.6. Preliminary Preparation

#### 2.6.1. Data Preparation and Preprocessing

Due to the difficulty in obtaining clinical data, we use Pommier’s overlapping chromosome datasets [46,47] to demonstrate the effectiveness of the present method. The dataset contains 13,434 overlapping chromosomes with a resolution of 94 × 93. For each image, there is a corresponding ground truth, in which each pixel represents an object class. In the segmentation map, class labels of 0, 1, 2, and 3 are denoted as the background (shown as black), non-overlapping regions of the first chromosome (shown as red), non-overlapping regions of the second chromosome (shown as green), and overlapping regions of chromosome (shown as blue), respectively. To match the images with our network, we padded the images to 128 × 128. The padding value of input images and ground truths was set as 255 and 0, respectively, to be consistent with the background of the original images. We divided the datasets into two subsets: 80% for training (a total number of 10,747 images) and the remaining 20% for testing (a total of 2686 images). In the training set, the number of pixels for the four classes were 167,373,977, 284,038, 5,138,621, and 1,282,212, respectively. In the testing set, the number of pixels for the four classes were 41,825,569, 575,220, 1,286,495, and 320,140, respectively. Therefore, we can calculate that the proportions of pixels for the four classes were 24.99%, 23.16%, 25.04%, and 24.97%, respectively. It shows that when we select 20% of images as testing set, there is no guarantee that every pixel class will be evenly divided. Moreover, the uneven distribution of pixel categories will affect the evaluation of classification results. Specifically, since some of the images in the testing set lack overlapping domains, this means that class 3 is missing. As a result, there is a clear bias in the actual final classification result. Therefore, in order to solve this problem and make our results reliable, we only kept pairs with the ground-truth containing overlapping domains for testing sets (a total of 2432 images).

#### 2.6.2. Implementation

In the training stage, all the training sets were shuffled, and all input images were normalized to the range of 0–1, and the batch size was set to 64. We optimized the generator and the discriminator alternately, both applying the Adam solver with a fixed learning rate of 0.0002 and momentum parameters of β1 = 0.5 and β2 = 0.999. Then, we set the random seed to 123. We trained our framework from scratch with the training sets to produce the “optimized” model. The training was stopped when training losses did not decrease for 200 consecutive epochs. We saved the generator model weights when the training Dice scores were at their highest. For the inference stage, we used the well-trained framework to segment the images. All the experiments were conducted in Pytorch [48] under an Ubuntu OS cloud server with an Intel Xeon(R) CPU E5-2680 v4 @2.40 GHz, 40 GB of RAM, and an NVIDIA Tesla P40 GPU with 24 GB of memory.

## 3. Results

### 3.1. Performance

**Figure 4:**
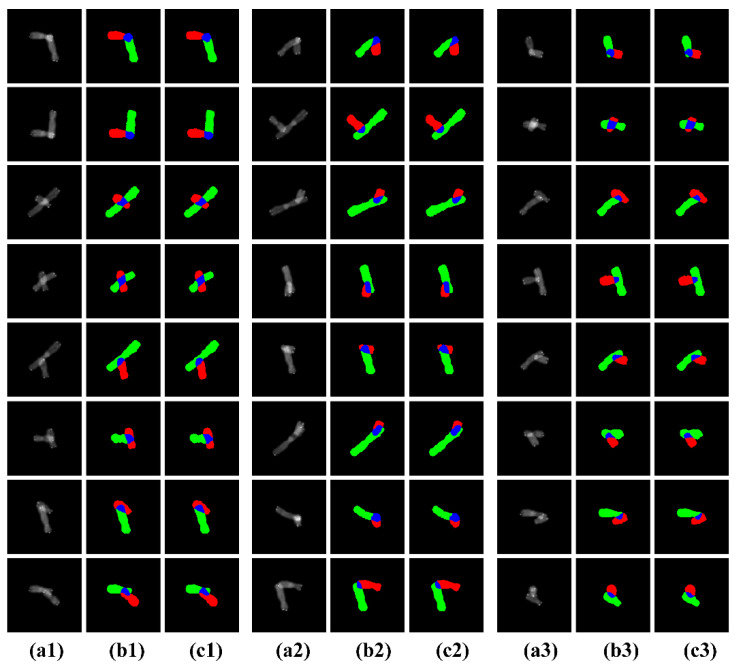
Some examples of segmentation results of the proposed method. The first to third, fourth to sixth, and seventh to ninth columns show the segmentation results of 24 chromosome images, respectively. (**a1**–**a3**) represent the source images, (**b1**–**b3**) represent the ground trut, (**c1**–**c3**) represent the segmentation result.

Figure 4 exhibits some examples of the segmentation results of our method, from which we can see that our method achieved an excellent visual perception result.

**Figure 5:**
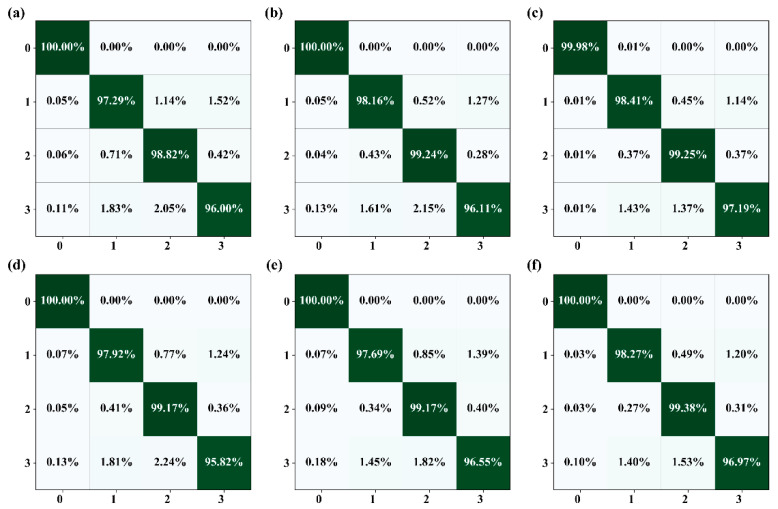
Confusion matrices of average accuracy scores on all the testing sets. Among them, (**a**) is the average each class IoU score of NestedUNet with CE loss, and (**b**–**f**) are our method with CE, Weight-CE, Dice, Weight-Dice, and Lovász-Softmax, respectively. For each image, the horizontal axis and the vertical axis represent predicted label and true label, respectively. The coordinate scale 0, 1, 2, and 3 represent the background non-overlapping regions of the first chromosome, non-overlapping regions of the second chromosome, and overlapping regions of chromosome, respectively. The entry in the *i*-th row and *j*-th column denotes the percentage of the testing samples from class *i* that were classified as class *j*.

Moreover, we can also see that the various scales of chromosome individuals and overlapping regions were correctly segmented in all the images, indicating that our method performed well in the multiscale segmentation task. In order to further highlight the superior performance of our algorithm, we show confusion matrices of average accuracy scores on all the testing sets in Figure 5. We can see that our method showed better results than other state-of-the-art methods. Through careful comparison, these quantitative results proved consistent with the quantitative results in Section 2.5, demonstrating the significant superiority of our method, not only for visual perception but also for quantitative analysis.

### 3.2. Performance Evaluation

#### 3.2.1. Visual Evaluation

**Figure 6:**
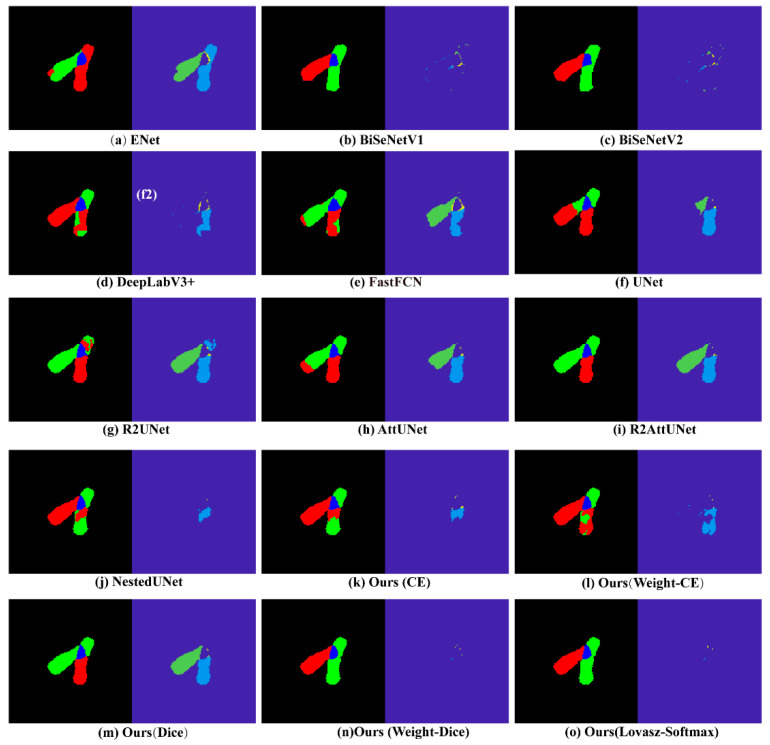
Examples of the corresponding segmentation results and difference images in pseudo-color map obtained by various methods. The left and right image of each example are the segmentation results and corresponding difference images, respectively. The different colors in pseudo-color map represent incorrect segmentation through the comparison with ground truth.

In this section, we visually compare the performance of our method with baseline methods. Figure 6 exhibits the results including difference images using pseudo-color map. Here, the difference images are generated through logical multiplication of the inversed ground truth and corresponding predicted result. Figure 6a–j show the results acquired using baseline methods with CE loss, while Figure 6k–o were acquired using the presented method with various loss functions. We can see from Figure 6 that our method with Lovász-Softmax or weight-dice loss achieved excellent segmentation results, while the performance of other methods was obviously poor, meaning that these methods do not learn effective features for the overlapping chromosome segmentation. NestedUNet performed the segmentation better than other large-scale models, indicating that multiscale feature learning is helpful for overlapping chromosome segmentation. Furthermore, we can see that the difference images acquired with our method were obviously cleaner than those acquired with other methods, indicating that the cGAN applied in our methods is effective to distinguish the segmented images and ground truths so as to better learn the features of the chromosomes. Additionally, it is clear that our method with Lovász-Softmax loss segmented the images more accurately, where almost every chromosomal region was correctly segmented, compared with other methods. This indicates that the Lovász-Softmax loss helped improve the discrimination ability of our method. In a word, the images shown in Figure 6 visually show that our AMFL framework can segment overlapping chromosomes with better performance than the baseline methods.

#### 3.2.2. Quantitative Evaluation

In this part, we quantitatively compare the performance of our method with others and show the results in Table 2. Here, we use the common CE loss for baseline methods. We can see that our AMFL achieved the best performance in all the metrics. This indicates that using cGAN to discriminate features can push the output distribution closer to ground truth so that our method outperforms others in overlapping chromosome segmentation tasks. It is also clear that the small-scale models presented almost the worst scoring in terms of Dice, IoU, and Hausdorff, while large-scale models reached better scores, suggesting that overlapping chromosome segmentation requires a more complex network structure. Again, NestedUNet achieved the top performance compared to other methods, quantitatively verifying the importance of multiscale feature analysis. It is worth emphasizing that our method had a lower Hausdorff distance score, indicating that it retained the shape and structure of the chromosome in the output images. The quantitative results, which are consistent with what we can see from Figure 6, prove the effectiveness of our method in overlapping chromosome segmentation.

Moreover, in order to further highlight the superior performances of our present method. In Table 3, we show the average IoU scores of each class and the accuracy for all the testing sets, compared against methods specifically designed for overlapping chromosome segmentation. We can see that our method has a significant advantage over these two methods in terms of the two quantitative metrics score. Especially for classes 1 and 2, IOU scores improved by 8.89% and 4.53%, respectively, over Hu et al.’s method and the IOU scores of all classes were better than those for CE-Net, which again proves the superiority of the AFML framework for overlapping chromosome segmentation. However, due to the imbalance of the categories in the training sets with a lack of overlapping areas, resulting in a slightly lower score of average IoU score for class 3 than Hu et al.’s method, but it was more in accordance with the diversity of clinical data. Since it is impossible that all chromosome images will have overlapping in the clinic, it is also very important to correctly separate nonoverlapping chromosomes.

#### 3.2.3. Computational Efficiency

To evaluate the computation efficiency, we present the total number of model parameters and the average running time of CPU and GPU when using different methods on all the testing sets in Table 4. The methods with small-scale networks consumed the least resources, obtaining the advancement of rate by sacrificing the accuracy. Among the methods with large-scale networks, our method took about 27 ms to segment an image on GPU, which ranked second only behind UNet; nevertheless, its model parameters were also the second smallest. Our method spent 568 ms on CPU, ranking fifth above R2UNet and R2AttUNet. The results show that, in addition to the outstanding segmentation performance, our method also performed well in computational efficiency, suggesting its great potential in real applications.

### 3.3. Ablation Study

In order to analyze the role of different parts of the proposed framework, we present the average quantitative results of the proposed method by using and without using GAN with different objective functions in Table 5. Obviously, the superiority of our method lies in the following: firstly, for the single model without using GAN, NestedUNet with Lovász-Softmax achieved an improved performance compared with other losses on all the testing sets. Secondly, the proposed AMFL adopted the GAN mechanism to discriminate features, resulting in a better scoring performance than the individual NestedUNet model without GAN. Thirdly, our method with Lovász-Softmax loss achieved the best performance in most of the metrics, demonstrating the effectiveness of using Lovász-Softmax to improve the discrimination ability. Fourthly, the framework with NestedUNet as the generator performed better than other configurations, indicating that modeling with multiscale features is effective for overlapping chromosome segmentation. Lastly, whether using or without using a GAN, the model with a NestedUNet as the backbone network performed better than other competitors. Moreover, we also split the dataset into training, validation, and testing sets, and we achieved results that were almost in close agreement with those of previous experiments. The Dice, IoU, and Hausdorff scores were 98.6163%, 97.6182%, 0.8293, respectively, demonstrating the reproducibility our results.

## 4. Conclusions

In this paper, we propose and demonstrate the AMFL framework for overlapping chromosome segmentation. In the network, instead of using single-scale features to represent chromosome images, we carefully designed a nested U-shaped network with dense skip connections as the generator to capture multiscale features to explore a better representation of the chromosome images. Then, we utilized cGAN to provide prior information for better discriminating features and producing highly accurate chromosome segmentation images. In addition, we replaced the common cross-entropy loss with the advanced Lovász-Softmax loss to improve the model’s optimization and accelerate the model’s convergence. In addition, we utilized the least-square GAN objective to replace the original GAN loss to stabilize the training and avoid model collapse. As for the objective function, we chose Lovász-Softmax after experimentally comparing it with others on their performance in overlapping chromosome segmentation. At last, to show the superiority of our AFML, we compared it with 10 state-of-the-art semantic segmentation methods. The results show that our AFML performed better in both visual perception and eight quantitative metrics.

Currently, our AFML performs well in public overlapping chromosome datasets. However, due to the difficulty in obtaining clinical data, our well-trained model may fail to meet situations where the images exist with severe morphological inconsistency in the clinical overlapping chromosome. In future research, we intend to collect annotated clinical data and design a generalized, fully automatic system for the segmentation, classification, and karyotype analysis of chromosomes.

## Figures and Tables

**Table 1:** The average quantitative results of proposed method by using different GAN generator.

G	Acc	Dice	IOU	Recall	Precision	FNR	FPR	Hausdorff
DeepLabV3+	99.9149	96.4414	93.8028	96.2027	96.9309	3.7973	0.3094	1.4366
FastFCN	99.9130	96.5195	93.8791	96.5454	96.7388	3.4546	0.2450	1.4376
UNet	99.9727	98.2346	96.9548	98.2836	98.3897	1.7164	0.0314	0.9524
R2UNet	99.9694	98.2646	97.0231	98.6001	98.1223	1.3999	0.0348	0.9423
AttUNet	99.971	98.2409	96.9582	98.2301	98.4575	1.7699	0.0304	0.9515
R2AttUNet	99.9585	97.8592	96.4989	98.1673	97.7693	1.8327	0.0399	1.0054
NestedUNet	99.9776	98.6048	97.5974	98.6550	98.7267	1.3450	0.0227	0.8252

Note that the units of all indicators are percentages except Hausdorff. A larger value of Acc, Dice, IoU, Recall, and Precision indicate better performance, while a smaller value of FNR, FPR, and Hausdorff shows a better performance. The best two results are highlighted in red and green, respectively.

**Table 2:** Average scores of various methods on eight metrics.

Method	Acc	Dice	IoU	Recall	Precision	FNR	FPR	Hausdorff
Small models								
ENet	99.8707	94.5821	90.7770	94.5365	95.0898	5.4635	0.3791	1.5861
BiSeNetV1	99.7361	90.9037	85.1075	89.3718	93.2404	10.6282	1.4966	1.9584
BiSeNetV2	99.8055	93.2973	88.8068	93.1980	93.8226	6.8020	0.6947	1.8145
Larger models								
DeepLabV3+	99.9048	95.8592	92.8454	96.0429	96.0126	3.9571	0.2623	1.4886
FastFCN	99.9170	96.4061	93.6931	96.6915	96.3868	3.3085	0.2017	1.4452
UNet	99.9684	97.8970	96.3765	97.9654	98.0156	2.0346	0.0331	1.0230
R2UNet	99.8659	95.1638	92.7348	96.0719	95.1458	3.9281	0.1046	1.2535
AttUNet	99.9625	97.6780	96.0418	97.7765	97.8637	2.2235	0.0395	1.0528
R2AttUNet	99.9122	95.7752	93.6760	96.6767	95.6791	3.3233	0.0792	1.1688
NestedUNet	99.9625	97.9670	96.6473	98.0266	98.0809	1.9734	0.0341	0.9518
**AMFL**	99.9776	98.6048	97.5974	98.6550	98.7267	1.345	0.0227	0.8252

Note that the units of all indicators are percentages except Hausdorff. A larger value of Acc, Dice, IoU, Recall, and Precision indicate a better performance, while a smaller value of FNR, FPR, and Hausdorff shows a better performance. The best two results are highlighted in red and green, respectively.

**Table 3:** Comparison of average IoU and accuracy scores for existing methods.

Method	Average IoU Scores				Accuracy
	Class 1	Class 2	Class 3	All Classes	
Hu et al. [22]	88.2	94.4	94.7	-	92.22
Hu et al. + TTA	-	-	-	-	99.27
Saleh et al. [23]	-	-	-	-	99.68
CE-Net [49]	96.04	97.76	90.35	-	99.92
U-Net-FIGI [50]	-	-	-	96.32	99.78
**AFML (Ours)**	97.09	98.93	94.37	97.60	99.98

Note that the index scores for all existing methods are drawn from the references, “-” indicates that it is not described in the paper. Class 1 and 2, are denoted as the two non-overlapping regions of the chromosomes and class 3 as the overlapping regions of chromosome. The best two results are highlighted in red and green, respectively. TTA indicates test time augmentation.

**Table 4:** The computational efficiency of various methods.

	**ENet**	**BiSeNet** **V1**	**BiSeNet** **V2**	**DeepLab** **V3+**	**FastFCN**	**UNet**
Params	0.35 M	12.43 M	2.85 M	59.46 M	104.3 M	34.53 M
GPU	63 ms	19 ms	34 ms	72 ms	78 ms	19 ms
CPU	38 ms	43 ms	34 ms	190 ms	380 ms	274 ms
	**R2UNet**	**AttUNet**	**R2AttUNet**	**NestedUNet**	**AMFL** **(Ours)**	**-**
Params	39.09 M	34.88 M	39.44 M	36.63 M	36.63 M	-
GPU	61 ms	27 ms	71 ms	27 ms	27 ms	-
CPU	715 ms	285 ms	734 ms	568 ms	568 ms	-

Params: The total number of model parameters. GPU/CPU: Average GPU/CPU runtime measured with reference to a full-resolution input (i.e., 128 × 128) on all the testing sets.

**Table 5:** The average quantitative results of the proposed method by using and without using GAN with different objective function.

Method	GAN	Acc	Dice	IOU	Recall	Precision	FNR	FPR	Hausdorff
NestedUNet with Dice	×	99.9457	97.3146	95.596	97.4631	97.3846	2.5369	0.0701	1.1617
NestedUNet with Weight-Dice	×	99.9434	97.1637	95.2851	97.3318	97.3021	2.6682	0.0929	1.2242
NestedUNet with CE	×	99.9625	97.9670	96.6473	98.0266	98.0809	1.9734	0.0341	0.9518
NestedUNet Weight-CE	×	99.9529	97.7494	96.0630	98.4318	97.3030	1.5682	0.0256	1.2219
**NestedUNet with Lovász-Softmax**	×	99.9714	98.2898	97.1699	98.3459	98.3993	1.6541	0.0326	0.9036
AMFL with Dice	√	99.9670	98.2422	97.0882	98.228	98.4479	1.772	0.0335	0.9166
AMFL with Weight-Dice	√	99.9699	98.3117	97.1692	98.3524	98.4454	1.6476	0.0381	0.9366
AMFL with CE	√	99.9735	98.4012	97.3453	98.3788	98.6395	1.6212	0.0266	0.8388
AMFL with Weight-CE	√	99.9699	98.4557	97.3259	98.7066	98.3769	1.2934	0.0175	0.9988
**AMFL with Lovász-Softmax**	√	99.9776	98.6048	97.5974	98.6550	98.7267	1.3450	0.0227	0.8252

Note that the units of all indicators are percentages except Hausdorff. A larger value of Acc, Dice, IoU, Recall, and Precision indicate a better performance, while a smaller value of FNR, FPR, and Hausdorff shows a better performance. The best two results are highlighted in red and green, respectively.

## Data Availability

The datasets generated and/or analyzed during the current study are available at https://www.kaggle.com/jeanpat/overlapping-chromosomes (accessed on 29 March 2022).

## References

[1] Basu A., Alder H., Khiyami A., Leahy P., Croce C.M., Haldar S. (2018). Down Syndrome and Micrornas. Biomed. Rep..

[2] Tramontana A., Hartmann B., Hafner E. (2019). DiGeorge syndrome chromosome region deletion and duplication: Prenatal genotype-phenotype variability in fetal ultrasound and MRI. Prenat. Diagn..

[3] Fang H., Liu S., Wang Y., Chiang C., Liu C., Lin C. (2019). Phenotypic features of a microdeletion in chromosome band 20p13: A case report and review of the literature. Mol. Genet. Genom. Med..

[4] Lin C., Zhao G., Yin A., Yang Z., Guo L., Chen H., Zhao L., Li S., Luo H., Ma Z. (2020). A novel chromosome cluster types identification method using ResNeXt WSL model. Med. Image Anal..

[5] Balagalla U.B., Samarabandu J., Subasinghe A. (2022). Automated human chromosome segmentation and feature extraction: Current trends and prospects. F1000Research.

[6] Madian N., Jayanthi K. (2014). Analysis of human chromosome classification using centromere position. Measurement.

[7] Minaee S., Fotouhi M., Khalaj B.H. A Geometric Approach for Fully Automatic Chromosome Segmentation. Proceedings of the IEEE Signal Processing in Medicine and Biology Symposium.

[8] Karvelis P., Tzallas A.T., Fotiadis D.I., Georgiou I. (2008). A Multichannel Watershed-Based Segmentation Method for Multispectral Chromosome Classification. IEEE Trans. Med. Imaging.

[9] Ritter G., Gao L. (2008). Automatic segmentation of metaphase cells based on global context and variant analysis. Pattern Recognit..

[10] Madian N., Jayanthi K.B. Overlapped Chromosome Segmentation And Separation Of Touching Chromosome For Automated Chromosome Classification. Proceedings of the 2012 Annual International Conference of the IEEE Engineering in Medicine and Biology Society.

[11] Saiyod S., Wayalun P., Sobecki J., Boonjing V., Chittayasothorn S. (2014). A New Technique for Edge Detection of Chromosome G-Band Images for Segmentation. Advanced Approaches to Intelligent Information and Database Systems.

[12] Poletti E., Zappelli F., Ruggeri A., Grisan E. (2012). A review of thresholding strategies applied to human chromosome segmentation. Comput. Methods Programs Biomed..

[13] Calzada-Navarrete V., Torres-Huitzil C. (2014). A local adaptive threshold approach to assist automatic chromosome image segmentation. Lat. Am. Appl. Res. Int. J..

[14] Bashmail R., Elrefaei L.A., Alhalabi W., Hassanien A., Tolba M., Shaalan K., Azar A. (2018). Automatic Segmentation of Chromosome Cells. Advances in Intelligent Systems and Computing.

[15] Gawande J.P., Manohar R., Gawande J.P., Manohar R., Gawande J.P., Manohar R. Watershed and Clustering Based Segmentation of Chromosome Images. Proceedings of the IEEE 7th International Advance Computing Conference.

[16] Sharma M., Saha O., Sriraman A., Hebbalaguppe R., Karande S. Crowdsourcing for Chromosome Segmentation and Deep Classification. Proceedings of the IEEE Conference on Computer Vision and Pattern Recognition Workshops.

[17] Lin C., Yin A., Wu Q., Chen H., Guo L., Zhao G., Fan X., Luo H., Tang H. Chromosome Cluster Identification Framework Based on Geometric Features and Machine Learning Algorithms. Proceedings of the IEEE International Conference on Bioinformatics and Biomedicine.

[18] Mei L., Guo X., Huang X., Weng Y., Liu S., Lei C. (2020). Dense Contour-Imbalance Aware framework for Colon Gland Instance Segmentation. Biomed. Signal Process. Control.

[19] Bhutto J.A., Tian L., Du Q., Sun Z., Yu L., Tahir M.F. (2022). CT and MRI Medical Image Fusion Using Noise-Removal and Contrast Enhancement Scheme with Convolutional Neural Network. Entropy.

[20] Pardo E., Morgado J.M.T., Malpica N. (2018). Semantic segmentation of mFISH images using convolutional networks. Cytom. Part A.

[21] Long J., Shelhamer E., Darrell T. Fully convolutional networks for semantic segmentation. Proceedings of the IEEE Conference on Computer Vision and Pattern Recognition.

[22] Saleh H.M., Saad N.H., Isa N.A.M. (2019). Overlapping Chromosome Segmentation using U-Net: Convolutional Networks with Test Time Augmentation. Procedia Comput. Sci..

[23] Hu R.L., Karnowski J., Fadely R., Pommier J.P. (2017). Image segmentation to distinguish between overlapping human chromosomes. arXiv.

[24] Chen P., Cai J., Yang L. Chromosome Segmentation via Data Simulation and Shape Learning. Proceedings of the 42nd Annual International Conference of the IEEE Engineering in Medicine & Biology Society (Embc).

[25] Altinsoy E., Yilmaz C., Wen J., Wu L., Yang J., Zhu Y., Rutkowski L., Scherer R., Korytkowski M., Pedrycz W., Tadeusiewicz R., Zurada J. (2019). Raw G-Band Chromosome Image Segmentation Using U-Net Based Neural Network. Artificial Intelligence and Soft Computing.

[26] Chen P., Li P., Li Q., Zhang D., Lia Q. (2019). Semi-Supervised Fine-Grained Image Categorization Using Transfer Learning with Hierarchical Multi-Scale Adversarial Networks. IEEE Access.

[27] Zhou Z., Siddiquee M.M.R., Tajbakhsh N., Liang J. (2020). UNet++: Redesigning Skip Connections to Exploit Multiscale Features in Image Segmentation. IEEE Trans. Med. Imaging.

[28] Isola P., Zhu J., Zhou T., Efros A.A. Image-To-Image Translation With Conditional Adversarial Networks. Proceedings of the IEEE Conference on Computer Vision and Pattern Recognition.

[29] Berman M., Triki A.R., Blaschko M.B. The Lov’asz-Softmax Loss: A Tractable Surrogate for The Optimization of the Intersection-Over-Union Measure in Neural Networks. Proceedings of the IEEE Conference on Computer Vision and Pattern Recognition.

[30] Ronneberger O., Fischer P., Brox T. U-Net: Convolutional Networks for Biomedical Image Segmentation. Proceedings of the Medical Image Computing and Computer-Assisted Intervention.

[31] Liu Y., Wang J., Qiu T., Qi W. (2021). An Adaptive Deblurring Vehicle Detection Method for High-Speed Moving Drones: Resistance to Shake. Entropy.

[32] Guo X., Nie R., Cao J., Zhou D., Mei L., He K. (2019). FuseGAN: Learning to Fuse Multi-Focus Image via Conditional Generative Adversarial Network. IEEE Trans. Multimed..

[33] Mao X., Li Q., Xie H., Lau R.Y.K., Wang Z., Smolley S.P. The Least Squares Generative Adversarial Networks. Proceedings of the International Conference on Computer Vision.

[34] Maas A.L., Hannun A.Y., Ng A.Y. The Rectifier Nonlinearities Improve Neural Network Acoustic Models. Proceedings of the International Conference on Machine Learning.

[35] Radford A., Metz L., Chintala S. (2015). Unsupervised Representation Learning with Deep Convolutional Generative Adversarial Networks. arXiv.

[36] Taha A.A., Hanbury A. (2015). Metrics for evaluating 3D medical image segmentation: Analysis, selection, and tool. BMC Med. Imaging.

[37] Sirinukunwattana K., Snead D., Rajpoot N.M. (2015). A Stochastic Polygons Model for Glandular Structures in Colon Histology Images. IEEE Trans. Med. Imaging.

[38] Paszke A., Chaurasia A., Kim S., Culurciello E. (2018). ENet: A Deep Neural Network Architecture for Real-Time Semantic Segmentation. arXiv.

[39] Yu C., Wang J., Peng C., Gao C., Yu G., Sang N. In Bisenet: Bilateral segmentation network for real-time semantic segmentation. Proceedings of the European Conference on Computer Vision (ECCV 2018).

[40] Yu C., Gao C., Wang J., Yu G., Shen C., Sang N. (2021). BiSeNet V2: Bilateral Network with Guided Aggregation for Real-Time Semantic Segmentation. Int. J. Comput. Vis..

[41] Chen L., Zhu Y., Papandreou G., Schroff F., Adam H. Encoder-Decoder with Atrous Separable Convolution For Semantic Image Segmentation. Proceedings of the European Conference on Computer Vision.

[42] Wu H., Zhang J., Huang K., Liang K., Yu Y. (2019). Fastfcn: Rethinking Dilated Convolution in the Backbone for Semantic Segmentation. arXiv.

[43] Alom Z., Yakopcic C., Hasan M., Taha T.M., Asari V.K. (2019). Recurrent residual U-Net for medical image segmentation. J. Med. Imaging.

[44] Oktay O., Schlemper J., Folgoc L.L., Lee M., Heinrich M., Misawa K., Mori K., Mcdonagh S., Hammerla N.Y., Kainz B. (2018). Attention U-Net: Learning Where To Look For The Pancreas. arXiv.

[45] Wang F., Jiang M., Qian C., Yang S., Li C., Zhang H., Wang X., Tang X. Residual Attention Network for Image Classification. Proceedings of the IEEE Conference on Computer Vision and Pattern Recognition.

[46] Pommier J. Overlapping Chromosomes. https://www.kaggle.com/jeanpat/overlapping-chromosomes.

[47] Pommier J. Overlapping Chromosomes. https://github.com/jeanpat/DeepFISH/tree/master/dataset.

[48] Paszke A., Gross S., Massa F., Lerer A., Chintala S. (2019). Pytorch: An imperative style, high-performance deep learning library. Adv. Neural Inf. Process. Syst..

[49] Gu Z., Cheng J., Fu H., Zhou K., Hao H., Zhao Y., Zhang T., Gao S., Liu J. (2019). CE-Net: Context Encoder Network for 2D Medical Image Segmentation. IEEE Trans. Med. Imaging.

[50] Sun X., Li J., Ma J., Xu H., Chen B., Zhang Y., Feng T. (2021). Segmentation of overlapping chromosome images using U-Net with improved dilated convolutions. J. Intell. Fuzzy Syst..

